# Fatty acid binding proteins (FABPs) in prostate, bladder and kidney cancer cell lines and the use of IL-FABP as survival predictor in patients with renal cell carcinoma

**DOI:** 10.1186/1471-2407-11-302

**Published:** 2011-07-18

**Authors:** Angelika Tölle, Saba Suhail, Monika Jung, Klaus Jung, Carsten Stephan

**Affiliations:** 1 Department of Urology, Charité - Universitätsmedizin Berlin, Berlin, Germany; 2 Berlin Institute for Urologic Research, Berlin, Germany

## Abstract

**Background:**

Fatty acid binding proteins (FABP) play an important role in carcinogenesis. Modified FABP expression patterns were described for prostate, bladder and for renal cell carcinoma. Studies on metabolic relationships and interactions in permanent cell lines allow a deeper insight into molecular processes. The aim of this study is therefore a systematic overview on mRNA and protein expressions of seven FABPs in frequently used urological cell lines.

**Methods:**

Nine cell lines of renal carcinomas, seven of urinary bladder carcinomas, and five of prostate carcinomas were investigated. Quantitative RT-qPCR and western blotting were used to determine different FABPs. In addition, 46 paired cancerous and noncancerous tissue samples from nephrectomy specimen with renal cell carcinomas were investigated regarding the ileum FABP mRNA expression level and associated with survival outcome.

**Results:**

General characteristics of all urological carcinoma cell lines were the expression of E-and IL-FABP on mRNA and protein level, while the expressions differed between the cell lines. The protein expression was not always congruent with the mRNA expression. Renal cell carcinoma cell lines showed expressions of L-, H- and B-FABP mRNA in addition to the general FABP expression in five out of the eight investigated cell lines. In bladder cancer cell lines, we additionally found the expression of A-FABP mRNA in six cell lines, while H-FABP was present only in three cell lines. In prostate cancer cell lines, a strong reduction of A- and E- FABP mRNA was observed. The expression of B-FABP mRNA and protein was observed only in the 22 RV-1 cells. IL-FABP mRNA was over-expressed in renal tumour tissue. The IL-FABP ratio was identified as an independent indicator of survival outcome.

**Conclusions:**

Distinctly different FABP expression patterns were observed not only between the cell lines derived from the three cancer types, but also between the cell lines from the same cancer. The FABP patterns in the cell lines do not always reflect the real situation in the tumours. These facts have to be considered in functional studies concerning the different FABPs.

## Background

Fatty acid binding proteins (FABPs) represent a family of cytosolic proteins containing 9 different types. The designations for the FABPs were derived from the tissue from which they had originally been isolated and include: (1) liver (L-FABP); (2) intestine (I-FABP); (3) heart (H-FABP); (4) adipocyte (A-FABP); (5) epidermis (E-FABP); (6) ileal (IL-FABP); (7) brain (B-FABP); (8) myelin (M-FABP) and (9) testis (T-FABP). The FABPs bind different fatty acids and take on the transport to cellular compartments. Thus, FABPs are involved in lipid metabolism and regulation of gene expression [1]. Recently, it has been shown that FABPs play an important role in carcinogenesis. Respective studies in solid tumours are summarised in table 1. Regarding urological tumours, modified FABP expression patterns were described for prostate [2] and urinary bladder cancer [3] as well as for renal cell carcinoma [4]. Hammamieh et al. [2] proved the influence of the FABPs on proliferation and apoptosis in prostate cancer. The loss of A-FABP expression in bladder carcinomas occurs mainly in invasive urothelial carcinomas [3] suggesting that FABPs could be used as tumour markers. In renal cell carcinomas, L- and H-FABP were decreased and B-FABP was up-regulated [4,5].

**Table 1:** Investigated FABP subtypes in solid carcinomas

FABP subtypes	Solid carcinomas
liver FABP (1)	colon [15], breast [16], kidney [4], liver [17], prostate [2], stomach [18],
intestine FABP (2)	colon [19], breast [16]
heart FABP (3)	kidney [5], stomach [20]
adipocyte FABP (4)	bladder [3], breast [16,21], prostate [2]
epidermal FABP (5)	bladder [14], breast [16], oral [22], pancreas [23], prostate [2]
ileum FABP (6)	colon [24]
brain FABP (7)	glioblastom [25,26], kidney [4], melanoma [27,28]

For studying metabolic relationship and interaction it is necessary to work with permanent cell lines derived from human tumours that allow a deeper insight into molecular processes. It is therefore the aim of this study to give a systematic overview on the mRNA and protein expression of seven FABPs in frequently used urological cell lines since information about FABPs in cell lines [2,6] is scarce and incomplete. In the present study, nine cell lines of renal carcinomas, seven of urinary bladder carcinomas and five of prostate carcinomas were included and present the essential object of our investigations. IL-FABP expression on transcript level in renal cell carcinoma tissues and its prognostic value is additionally given as preliminary study for the use of FABPs in clinical research. We decided to study renal cell carcinoma as example for clinical specimens, since our group have already investigated in previous studies [4] the expression of the two FABP-types B-FABP and L-FABP in renal cell carcinomas.

## Methods

### Cell culture

Cell lines originating from renal cell carcinoma, bladder and prostate cancer representing the three most frequent urologic carcinomas were included in the study (Table 2). Cells purchased from American Type Culture Collection (Manassas, VA USA) and from German Collection of Microorganism and Cell Culture (Braunschweig, Germany) were cultured as described in the manufacturer's instructions. The LNCaP and SW-839 cells were cultured in Primaria flasks (Becton, Dickinson and Company, Heidelberg, Germany) and all the other cells in standard usual flasks (Becton, Dickinson and Company) maintained in a humid chamber at 37°C and 5% CO2. All cell lines except Caki-1, Caki-2, SW 839 were maintained in RPMI 1640 medium. Caki-1, Caki-2 and SW 839, ACHN and HK-2 were maintained in McCoy's 5A medium, the cell line ACHN in MEM medium and the HK-2 cells in K-SFM medium. All tissue culture media were obtained from Invitrogen GmbH (Karlsruhe, Germany) and fetal calf serum from PAA Laboratories GmbH (Pasching, Austria). The media were supplemented with antibiotics (1 × penicillin/streptomycin: 100 U/ml and 100 μg/ml, PAA Laboratories). All cells were harvested under microscopic control with a solution of 0.5 g/l trypsin plus 0.2 g/l EDTA (Invitrogen GmbH) solved in PBS and frequently washed and counted in Dulbecco's PBS from PAA Laboratories.

**Table 2:** Examined cell lines of urological carcinomas in this study

Renal cell carcinoma		Prostate carcinoma		Urinary bladder carcinoma	
Cell line	Origin	Cell line	Origin	Cell line	Origin
786-0	Primary adenocarcinoma	BPH-1	Benign hyperplasia	HCV-29	Normal bladder irradiated
A 498	Primary tumour	DU-145	Metastasis of brain	HT-1376	Primary transitional cell carcinoma G3
A 704	Primary adenocarcinoma	LNCaP	Metastasis of lymph node	J-82	Primary transitional cell Carcinoma
ACHN	Primary adenocarcinoma	PC-3	Metastasis of bone marrow	RT-4	Primary transitional cell carcinoma T2,G1
Caki-1	Clear cell carcinoma Metastasis of skin	22 RV-1	Primary tumour	RT-112	Primary transitional Cell carcinoma G2
Caki-2	Primary clear cell carcinoma			SCaBER	Primary squamous Cell carcinoma
HK-2	Normal tissue proximal tubule			UM-UC-3	Primary transitional cell carcinoma
SN-12	Primary tumour				
SW 839	Primary tumour				

### Tumour material and clinico-pathological data of patients

Forty-six matched (malignant and non-malignant) kidney specimens from primary tumours were used for total RNA isolation. The samples were derived from patients with renal cell carcinomas (RCC) undergoing radical tumour nephrectomy at the Department of Urology, Charité - Universitätsmedizin Berlin, Germany between 2003 and 2005. Staging met the UICC 2002 criteria. Histological classification was performed according to the WHO criteria; tumour grading was accomplished according to Fuhrman. The clinico-pathological parameters in all RCC were summarised in table 3. The endpoint of the survival analyses was the cancer-related survival measured from the date of the radical nephrectomy to the time of the last follow-up or death. This study has been approved by the Charité University Ethics Committee.

**Table 3:** Clinico-pathological characteristics of the study cohort of patients with renal cell carcinomas

		Total	Clear cell RCC	papillary RCC	Other** RCC
All cases		46	36	2	8
Age, years*		40 - 78			
Median*		61.0			
Sex	male	35			
	female	11			
Grading	G1	3	3		
	G2	30	27	2	1
	G3	11	6		5
	G4	2			2
pT State	pT1a	6	6		
	pT1b	8	6		2
	pT2	2	2		
	pT3a	11	8		3
	pT3b	15	11	2	2
	pT3c	2	2		
	pT4	2	1		1
Metastasis	M0	27	24	1	2
	M1	19	12	1	6
Lymph nodes	N0	14	14		
	N1	9	4	2	3
	NX	23	18		5

* Values are given as range and median

** This group involves six sarcomatoide, one mixed form (clear cell/papillary) and one Ductus Bellini carcinoma

### RNA isolation from cells and tissue samples

The total RNA from cells and tissue material were isolated using the RNeasy mini Kit from Qiagen (Qiagen GmbH, Hilden, Germany). The cell number was adjusted between 2 and 6 × 10^6 ^cells. All preparation steps were performed according to the supplier's protocol. The sample pairs from the same kidney were collected immediately after surgery in RNAlater^® ^Stabilization Reagent (Qiagen, Hilden, Germany), stored overnight at 4°C and at -80°C afterwards. The RNA isolation was performed as formerly described by our group [4] and resulted in RNA samples with an absorbance of 260/280 nm ratio in the range from 1.96 to 2.08 and RNA integrity values of 9.7 ± 0.8 (mean ± SD) for renal, of 9.9 ± 0.22 for prostate and of 10.0 ± 0.08 for bladder cell lines as well as of 8.4 ± 1.02 for tissue samples.

### Real-Time PCR

One μg RNA of the various urological cell lines and of the kidney tissues respectively, was reverse transcribed using the Transcriptor First Strand cDNA Synthesis Kit (Roche Applied Science, Mannheim, Germany) by random hexamer priming method according to the manufacturer's recommendations. The detailed procedure was previously described [4]. All used primers and probes were summarised in table 4.

**Table 4:** Sequences of primer sets and UPL probes

Gene	Accession number	Primer	Sequences (5'...3')	Product size[bp]	UPL probe*
L-FABP1	NM_001443	Forward	ttctccggcaagtaccaact	93	72
		Reverse	cttccccttctggatgagc		
I-FABP2	NM_000134	Forward	acaacctagcagacggaactg	78	1
		Reverse	tccgtttgaattttccaataagtt		
H-FABP3	NM_004102	Forward	ctgggcacctggaagcta	77	56
		Reverse	ctggtagcaaaacccacacc		
A-FABP4	NM_001442	Forward	cctttaaaaatactgagatttccttca	105	72
		Reverse	ggacacccccatctaaggtt		
E-FABP5	NM_001444	Forward	gcagacccctctctgcac	138	56
		Reverse	tcgcaaagctattcccactc		
IL-FABP6	NM_001445	Forward	ctcagagatcgtgggtgaca	68	22
		Reverse	tcacgcgctcataggtca		
B-FABP7	NM_001446	Forward	ctcagcacattcaagaacacg	69	33
		Reverse	ccatccaggctaacaacagac		
TBP	NM_003194	Forward	ttcggagagttctgggattgta	227	
		Reverse	tggactgttcttcactcttggc		
			Probe: **F**-ccgtggttcgtggctctcttatcctcaT-**P**		
PPIA	NM_021130	Forward	Hs_PPIA_I_SG	121	
		Reverse	QuantiTect Primer Assay		
			Qiagen (Cat. No. QT00052311)		

* UPL probes from Universal ProbeLibrary; Roche

**Figure 1:**
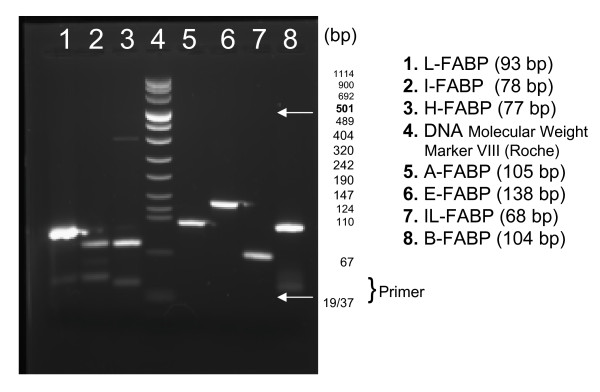
**A submarine agarose gel electrophoresis  for PCR product control**. The PCR amplificates run in 3% (w/v) agarose, small DNA low melt (Biozym, Oldendorf, Germany) in TBE buffer pH 8 and ethidium bromide (0.5 μg/ml).

The previously characterised conditions [4] were used for all materials and for all FABP types except A-FABP, for which an annealing temperature of 58°C for 20 s was preferred. For relative quantification of FABP mRNA expression, we also determined the two genes TATA box-binding protein (TBP) and peptidylproline isomerase A (PPIA) verified in a previous study as most suitable reference genes for gene profiling studies in renal cell carcinoma tissue [7]. Standard curves were generated for all gene-specific PCRs with cDNA samples from cell lines or tissues to calculate gene expression and PCR efficiencies that were between 1.85 and 2.01. The specificity of PCR was checked by the length of PCR products using agarose gel electrophoresis (Figure 1). All samples were measured in duplicates. The intraserial run precision (n = 9) of the PCR measurements, measured by the determination of IL-FABP amounted to a variation coefficient of 6.62% at a mean concentration of 2.66 × 10^-2 ^(Cq-value of 26.09).

### Protein extraction and western blot analysis

Suspensions of 2 - 3 × 10^7 ^cells were washed two times in PBS and then sedimented by 300 × g centrifugation for 10 min. Cell lysates were prepared according to the method of Zigrino et al. [8]. Protein extraction of paired tissue samples (carcinoma/normal) from six nephrectomy specimens was carried out as described by Fritzsche et al. [9]. All protein extracts were run under reducing conditions in a 15% SDS-polyacrylamide gel. Western blot analysis was carried out using the standard protocol for protein transfer onto PVDF membrane (Millipore Corp, Bedford, MA, USA) according to Towbin et al. [10]. Western blotting was performed with FABP type specific antibodies as summarised in table 5. Actin served as loading control. The antigen-antibody reaction was visualized by ECL Advance™ Western Blotting Detection Kit (GE Healthcare UK Limited, Little Chalfont Buckinghamshire, UK). Intensity of the detected signals by Western blot was quantified with Fluor-S MultiImager (Bio-Rad Laboratories, Hercules, USA).

**Table 5:** Antibodies for Western Blot detection

Protein	Species		Supplier	Concentration
L-FABP	Rabbit IgG	polyclonal	Hycult biotechnology b.v., Uden, Netherlands	1 μg/ml
I-FABP	Rabbit IgG	polyclonal	Hycult biotechnology b.v., Uden, Netherlands	2 μg/ml
H-FABP	Mouse IgG_1_	monoclonal Clone 66E2	Hycult biotechnology b.v., Uden, Netherlands	1 μg/ml
A-FABP	Rabbit IgG	polyclonal	Hycult biotechnology b.v., Uden, Netherlands	0.2 μg/ml
E-FABP	Rabbit IgG	polyclonal	Hycult biotechnology b.v., Uden, Netherlands	0.1 μg/ml
IL-FABP	Rabbit IgG	polyclonal	Hycult biotechnology b.v., Uden, Netherlands	1 μg/ml
B-FABP	Rabbit IgG	polyclonal	Hycult biotechnology b.v., Uden, Netherlands	1 μg/ml
β-actin	Mouse IgG_2_	monoclonal Clone AC-74	Sigma-Aldrich Chemie GmbH, Munich, Germany	2.1 μg/ml
anti-rabbit IgG/HRP*	Goat	polyclonal	DakoCytomation, Glostrup, Denmark	0.12 μg/ml
anti-mouse IgG/HRP*	Rabbit	polyclonal	DakoCytomation, Glostrup, Denmark	0.53 μg/ml

* HRP - Horseradish peroxidase

### Statistical analysis

Statistical analysis was performed with SPSS, version 19.0 (SPSS Inc, Chicago, IL, USA). P values < 0.05 were considered significant.

## Results

### FABP expression on transcript and protein levels in cell lines

**Figure 2:**
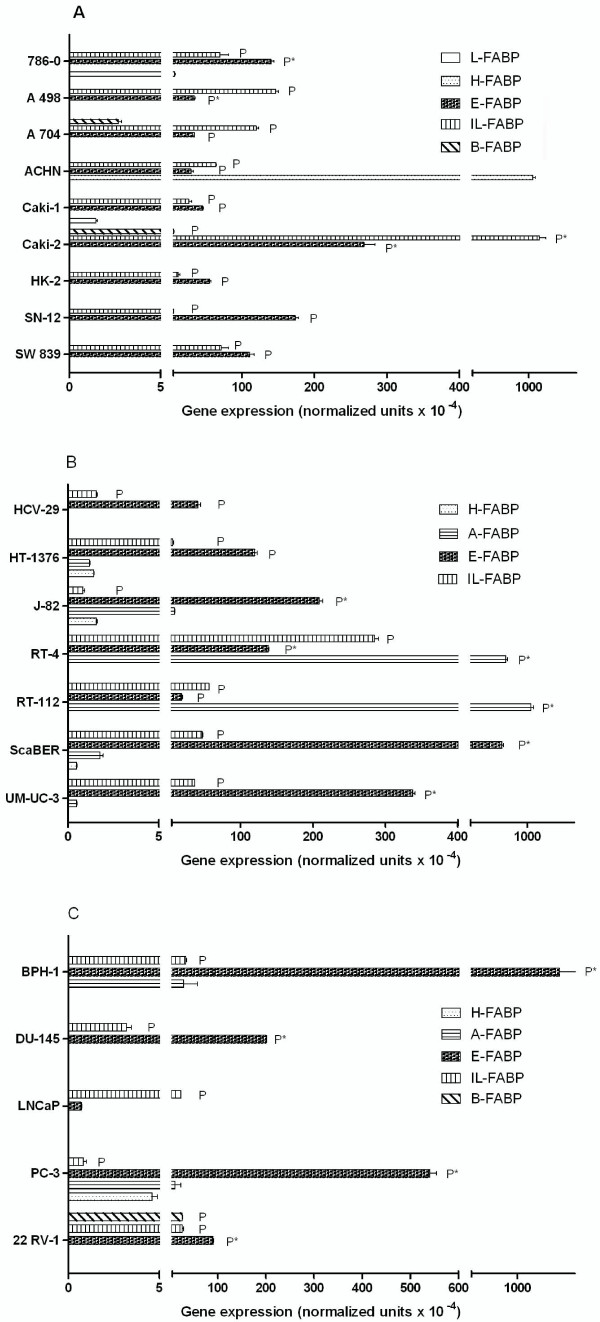
**FABP subtype mRNA expression in the various carcinoma cell lines**. (A) Renal cell carcinoma cell lines; (B) Urinary bladder carcinoma cell lines; (C) Prostate carcinoma cell lines. P labelled the immunoblot detection of FABP and P* marked a very strong immunoblot detection of FABP.

The general characteristic of all urological carcinoma cell lines is the expression of E-and IL-FABP both on mRNA and protein level (Figure 2A-C), while the expressions differed between the cell lines. In addition, in all cell lines studied neither the I-FABP protein nor the I-FABP mRNA was detectable.

### Renal cell carcinoma cell lines

The cell line HK-2 derived from normal renal tissue only expressed mRNAs of E-FABP and IL-FABP. Its mRNA expression pattern differed in comparison to the five carcinoma cell lines 786-0, A 704, ACHN, Caki-1 and Caki-2, which showed additional FABPs. The 786-0 and Caki-1 cells expressed L-FABP, ACHN cells expressed H-FABP and A 704 and Caki-2 cells expressed B-FABP.

**Figure 3:**
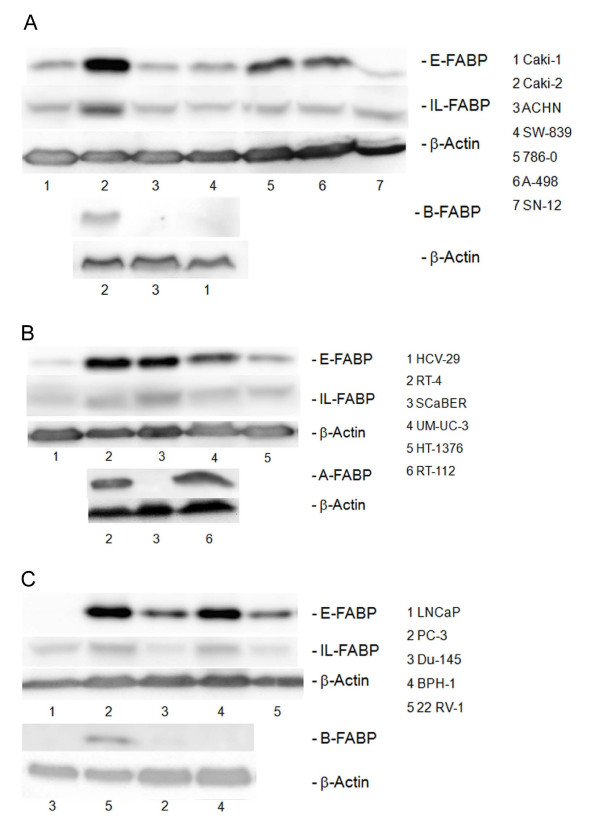
**Western blot detection of FABP subtypes in various carcinoma cell lines**. Exemplary protein detection in (A) renal carcinoma cells in (B) urinary bladder carcinoma cells and in (C) prostate carcinoma cells.

The protein expression was not always congruent with the mRNA expression and the levels of E- and IL-FABP in these cells were different in the cell lines. In 786-0, A 498 and Caki-2 cells, the E-FABP protein expression was very high (Figure 3A). Caki-2 cells were characterised also by a high IL-FABP level. All the other mRNAs except B-FABP in Caki-2 cells (Figure 2A) were not transcribed to the corresponding protein that was not detectable at all.

### Urinary bladder carcinoma cell lines

E- and IL-FABP were ubiquitously detectable as mRNA and protein in all bladder cell lines (Figure 2B). The protein expression level was increased in the carcinoma lines in comparison to normal bladder cell line HCV-29 (Figure 3B). The carcinoma cell lines were characterised by the two additional H- and A-FABP mRNAs. The mRNA and protein expression was not always congruent. The HT-1376, J-82 and SCaBER cells expressed A-FABP mRNA, but the protein was not detectable. The situation for the H-FABP mRNA was similar. The highest A-FABP mRNA expression was observed in RT-4 and RT-112 cells and here the protein was also detectable (Figure 3B).

### Prostate carcinoma cell lines

E-FABP was found to be the main FABP in prostate carcinoma cell lines (Figure 2C), while the LNCaP cell lines showed the lowest mRNA expression and the protein expression was lacking (Figure 3C). The expression of IL-FABP mRNA was lower compared to the E-FABP mRNA. The protein detection of IL-FABP was positive in all cell lines, but distinct intensity differences were observed. In DU-145 and 22 RV-1 cells, the protein content was lower than in the other cell lines (Figure 3C).

The benign prostate hyperplasia cell line (BPH-1) was characterised by the two additional mRNAs of H-FABP and A-FABP. Two cell lines derived from metastasis, DU-145 and LNCaP, lost of these two mRNAs (H-FABP and A-FABP). The 22 RV-1 cells, derived from a primary prostate carcinoma, were marked by the loss of A-FABP mRNA and appearance of B-FABP mRNA and the corresponding protein. Protein detection of H- and A-FABP was not possible in all cell lines, so that differences between mRNA and protein expression appeared there, too.

### IL-FABP mRNA expression in RCC tissue

**Figure 4:**
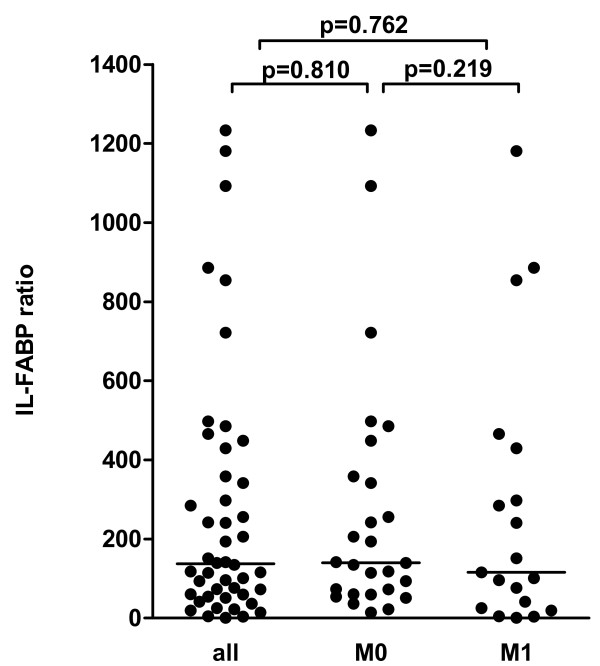
**IL-FABP ratio in a cohort of patients with renal cell carcinoma**. The IL-FABP ratio was calculated as relation of normalised IL-FABP mRNA concentration in tumour tissue to normalised concentration in normal tissue. Expression data were normalised against the geometric means of the two reference genes PPIA and TBP. The median of IL-FABP ratio was calculated for all patients, for Patients without metastasis (M0) and for patients with metastasis (M1): Statistical differences were calculated by the Mann Whitney test.

The expression of IL-FABP in normal and tumour tissues were normalised against the geometric mean expression of the two reference genes PPIA and TBP. The increase of IL-FABP in the tumour tissues was calculated as relation of normalised concentration in tumour tissue to normalised concentration in normal tissue and further on mentioned as ratio. The IL-FABP mRNA expression was 137-times higher (median) in RCC than in the normal tissue (p < 0.0001; Figure 4). The IL-FABP ratio was not correlated to the tumour grade and stage (r_s _= -0.143, p = 0.343; r_s _= -0.181, p = 0.228). Samples from patients without metastasis at the time of radical nephrectomy (27 cases) displayed a 139-fold (median) over-expression (Figure 4), while samples from nephrectomised patients with metastases showed 116-fold (median) over-expression (Figure 4). This difference was not significant (p = 0.219).

### IL-FABP expression and patient survival time

**Figure 5:**
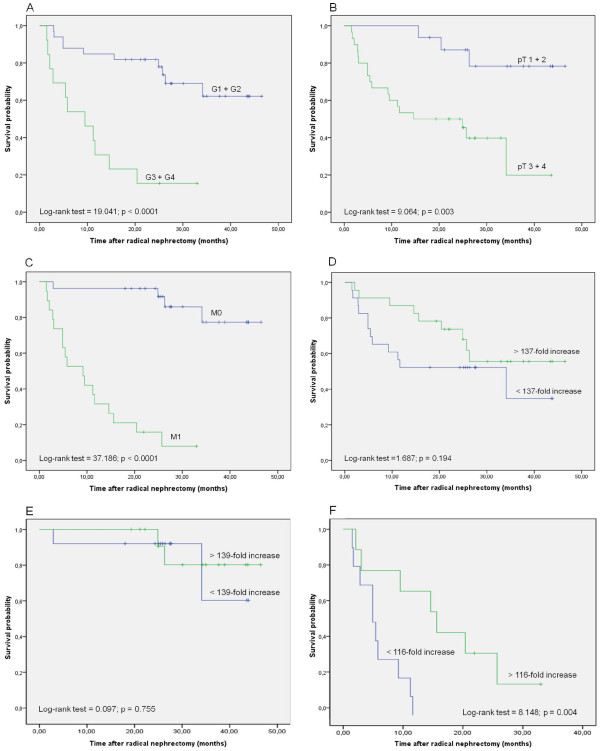
**Kaplan-Meier survival curves for IL-FABP mRNA expression in matched RCC tissue samples**. (A) Fuhrman grades 1 and 2 compared to 3 and 4, (B) Tumour stage pT1 and pT2 (tumour is limited to the kidney) compared to pT3 and pT4 (tumour extends over the organ), (C) Patients without metastasis (M0) compared to those with metastasis (M1), (D) IL-FABP ratio above and below the median was compared for all cases, (E) IL-FABP ratio above and below the median was compared in the group of patients without metastasis (M0), (F) IL-FABP ratio above and below the median was compared in the group of patients with metastasis (M1).

The tumour cohort studied was representative because the conventional prognostic parameters (tumour stage, tumour grade and metastasis) reached significance for survival in Kaplan-Meier analysis (Figure 5). Patients with high-stage, high-grade tumours and with metastasis had a lower cancer-related survival time. Taking all patients together, the IL-FABP ratio in the tumour tissue was not associated with the survival time (log rank test, p = 0.194). However, differences were found when the two subgroups of patients with and without metastasis at the date of radical nephrectomy were separately analysed. In patients without metastasis, no association was found between the IL-FABP ratio and the survival time (log-rank test, p = 0.755). In contrast, in the group of patients with distant metastases (M1; n = 19), an increased ratio of IL-FABP in the malignant compared with the non-malignant tissue sample was significantly associated with a longer survival time (log-rank test, p = 0.004; Figure 5C). Using the multivariable Cox regression analysis with the variables pT stage, grade, metastasis and the mentioned IL-FABP ratio, IL-FABP arose as independent variable (log-rank test, p = 0.029). This result was internally validated with a bootstrap analysis with 5000 bootstrap replicates and the 95% percentile approach. The independence of the variable IL-FABP ratio was confirmed (log-rank test, p = 0.026).

## Discussion

Approximately one quarter of all newly diagnosed cancer patients have urological tumours [11]. Thus, the purpose to improve early diagnosis and to introduce new reliable therapies for these cancer patients is a main objective of urological research. It is based on the search for characteristic tissue markers that can be used not only as diagnostic and/or prognostic biomarkers but also as targets for new treatment strategies. In this respect, the altered expression of FABPs in solid tumours and especially in urological tumours is of great interest as briefly discussed in the section Background (Table 1). The observation of altered expression is to be verified under experimental conditions and it is necessary to work with well described permanent cell lines relating to the FABP expression. To the best of our knowledge we present in this study the first systematic investigation concerning the expression of most FABPs in urological cell lines.

Our data show three essential features: (a) the expression of E- and IL-FABP and the lack of expression of I-FABP on mRNA and protein levels in all urological cell lines (Figure 2A-C), (b) the inconsistent congruence between the mRNA expression and protein expression differing both between the various FABPs and the cell lines as described in detail in the section Results and (c) the different patterns of FABPs not only between but also within the organ-specific cell lines.

Discrepancies between RNA and protein could be explained by different analytical sensitivities of the methods, in case of RT-PCR and Western blot technique, and more probably, by biological reasons. As extensive cellular machinery is involved in the process of translation, possible differences between RNA and protein expression can be caused by posttranscriptional regulatory mechanisms. These processes are still poorly understood. One possibility is that small non-coding RNAs might influence the translation.

In addition to the general expression of E- and IL-FABP in all cell lines, five out of the eight investigated renal cell carcinoma cell lines showed expressions of L-, H- and B-FABP mRNA. Only the Caki-2 cells represented the situation in renal cell carcinoma tissue, particularly the decreased or absent expression of L- and H-FABP and the up-regulation of B-FABP mRNA. Protein of B-FABP was exclusively detectable in the Caki-2 cells. It was known from earlier studies [4,5] that renal tumour tissue showed reduced expression levels of L-FABP and H-FABP and increased B-FABP compared to normal renal tissues.

The results of IL-FABP expression and our previous observations regarding the clinical significance of B- and L-FABPs in renal cell carcinoma [4] prompted us to examine IL-FABP mRNA expression in matched malignant and non-malignant specimens from kidney after radical nephrectomy. IL-FABP mRNA was over-expressed in the tumour tissue and no statistical differences were observed between samples from patients with and without metastases at the date of surgery. However, it was remarkable that patients with metastasis and an increased IL-FABP mRNA ratio showed a longer survival time. The IL-FABP ratio was identified as an independent indicator for survival outcome. Along with the retrospective nature of this study, the low number of patients was a limitation of this investigation. However, the internal validation using a bootstrap approach proved this result despite the low number of patients investigated. Therefore, we consider this as preliminary result that would be worth to be confirmed in further studies. Patients with high grade tumours and metastasis have a poor prognosis. In this group, a high IL-FABP expression might be an advantage because the transport of fatty acids could be enhanced. It is a speculation that cells incorporate a large amount of long-chain polyunsaturated fatty acids and so the level of antioxidants could be increased in the cells. This could be a protection against elevated ROS level in cancer cells.

In summary, this and the former study showed that the renal cell carcinoma tissue was characterised by decreased L- and H-FABPs and increased B- and IL-FABPs. The changes of these four FABP types indicate fundamental alterations in the fatty acid metabolism in the RCC carcinogenesis. Most of the investigated renal carcinoma cell lines only partly reflected these changes in tumour tissue.

In bladder cancer cell lines, we found in addition to the general expression of E- and IL-FABP the expression of A-FABP in six examined cell lines, while H-FABP was present only in three cell lines (HT-1376, J-82, SCaBER). A high expression of A-FABP mRNA and protein was observed in the well-differentiated RT-4 and RT-112 cells, while the other cell lines, which had been derived from less-differentiated, high-grade bladder tumours such as HT-1376 cells showed lower A-FABP mRNA expression. This result corresponds to data of Ohlsson et al. [3] and Boiteux et al. [12] who described the loss of A-FABP as marker for bladder cancer progression. On the other hand, the E-FABP mRNA expression in the HT-1376 cells, derived from a poorly differentiated G3 tumour [13], was higher than in the well-differentiated RT-112 cells, whereas Ostergaard et al. [14] reported a decreased expression of E-FABP in less-differentiated tumours. Thus, this result also supports the conclusion that the expression in the cells does not always reflect the situation in tumours.

The expression pattern of various FABP types in prostate cancer cell lines was previously described by Das et al. [6]. The authors observed a strong reduction of A-, E- and H-FABP in the cancer cell lines LNCaP, DU-145, and PC-3 compared to cells from normal primary cell cultures. The present work proved this result for A- and E-FABP in comparison to the BPH-1 cells derived from benign prostate hyperplasia. We observed in androgen-sensitive LNCaP cells a more than 1500-fold lower expression of E-FABP than in androgen-insensitive BPH-1. The expression of B-FABP mRNA and protein was observed only in the 22 RV-1 cells derived from a primary tumour. Das et al. [6] observed B-FABP in LNCaP cells and in prostate cancer tissues of well-differentiated tumours and suggested B-FABP as stage-specific marker in prostate cancer. L-and I-FABP were not detected in the present study at all. LNCaP cells were characterised by IL-FABP and a low expression of E-FABP mRNA.

## Conclusions

Our study is the first report about the mRNA and protein expression of various FABP types in frequently used cell lines from prostate, bladder, and kidney cancers. Regarding to carcinoma cell lines, distinct differences of FABP expression patterns were observed between the cells derived from the three cancer types but also between the organ-specific cell lines. In addition, the FABP patterns in the cell lines do not reflect the real situation in the tumours. It was suggested that in normal tissues a balance exists between different FABP types and that this balance is destroyed in cancerogenesis causing a new composition of FABP types [6]. To study this effect in more detail, the knowledge on the FABP expression as given in this study might be helpful in selecting an appropriate cell line for experimental work.

## Competing interests

The authors declare that they have no competing interests.

## Authors' contributions

AT coordinated the study, performed statistical analyses and wrote the paper. SS performed the RT-PCR and western blots. MJ realised the primer design and controlled the quality of PCR reactions. KJ supported statistical analyses and revised the paper. CS provided samples and clinico-pathological data. All authors read and approved the final manuscript.

## Pre-publication history

The pre-publication history for this paper can be accessed here:

http://www.biomedcentral.com/1471-2407/11/302/prepub
